# Whole-genome sequencing of Atacama skeleton shows novel mutations linked with dysplasia

**DOI:** 10.1101/gr.223693.117

**Published:** 2018-04

**Authors:** Sanchita Bhattacharya, Jian Li, Alexandra Sockell, Matthew J. Kan, Felice A. Bava, Shann-Ching Chen, María C. Ávila-Arcos, Xuhuai Ji, Emery Smith, Narges B. Asadi, Ralph S. Lachman, Hugo Y.K. Lam, Carlos D. Bustamante, Atul J. Butte, Garry P. Nolan

**Affiliations:** 1 Institute for Computational Health Sciences, University of California San Francisco, San Francisco, California 94158, USA;; 2 Roche Sequencing Solutions, Belmont, California 94002, USA;; 3 Department of Genetics, Stanford University School of Medicine, Stanford, California 94305, USA;; 4 Baxter Laboratory for Stem Cell Biology, Department of Microbiology and Immunology, Stanford University, Stanford, California 94305, USA;; 5 International Laboratory for Human Genome Research, National Autonomous University of Mexico (UNAM) Santiago de Querétaro, Querétaro 76230, Mexico;; 6 Human Immune Monitoring Center and Functional Genomics Facility, Stanford University, Stanford, California 94305, USA;; 7 Ultra Intelligence Corporation, Boulder, Colorado 80301, USA;; 8 Department of Pediatric Radiology, Stanford University School of Medicine, Stanford, California 94305, USA

## Abstract

Over a decade ago, the Atacama humanoid skeleton (Ata) was discovered in the Atacama region of Chile. The Ata specimen carried a strange phenotype—6-in stature, fewer than expected ribs, elongated cranium, and accelerated bone age—leading to speculation that this was a preserved nonhuman primate, human fetus harboring genetic mutations, or even an extraterrestrial. We previously reported that it was human by DNA analysis with an estimated bone age of about 6–8 yr at the time of demise. To determine the possible genetic drivers of the observed morphology, DNA from the specimen was subjected to whole-genome sequencing using the Illumina HiSeq platform with an average 11.5× coverage of 101-bp, paired-end reads. In total, 3,356,569 single nucleotide variations (SNVs) were found as compared to the human reference genome, 518,365 insertions and deletions (indels), and 1047 structural variations (SVs) were detected. Here, we present the detailed whole-genome analysis showing that Ata is a female of human origin, likely of Chilean descent, and its genome harbors mutations in genes (*COL1A1*, *COL2A1*, *KMT2D*, *FLNB*, *ATR*, *TRIP11*, *PCNT*) previously linked with diseases of small stature, rib anomalies, cranial malformations, premature joint fusion, and osteochondrodysplasia (also known as skeletal dysplasia). Together, these findings provide a molecular characterization of Ata's peculiar phenotype, which likely results from multiple known and novel putative gene mutations affecting bone development and ossification.

In 2003, the Atacama humanoid skeleton (Ata) was discovered in a deserted mining town La Noria in the Atacama region of Chile. The Ata specimen had multiple abnormalities and unusual features, including a height of 6 in, a skull with signs of turricephaly (high-head syndrome, a birth defect in which the top of the skull is cone-shaped), fewer than expected number of ribs, and apparently prematurely ossified growth plates, suggesting a greater age at time of death than the size of the specimen would indicate. This led to speculation that this was either a preserved nonhuman primate, human fetus harboring genetic mutations, or a preterm infant with birth defects. Furthermore, Ata was also featured in a documentary titled *Sirius*, in which it was hypothesized that this specimen was a preserved humanoid or possible evidence of alien life (Greer et al. 2013).

To better understand the origins of this specimen, an analysis was initiated in 2012. Although the Ata specimen was hypothesized to be ancient, multiple analyses and unpublished reports collectively implied that the specimen was not ancient, but belonged to the modern age, and contained high-quality DNA that was suitable for scientific investigation. A series of unpublished analyses were performed using skeletal radiography, computed tomography (CT), and whole-genome sequencing, and our preliminary investigation revealed that the specimen was indeed human (Supplemental Note). Further, through DNA analyses, we found that the Ata specimen's mitochondrial B2 haplotype group had significant overlap with South American population. After examining the X-ray images, it was concluded that Ata had only 10 pairs of ribs instead of the normal 12 in humans, and Ata's estimated bone age suggested by precocious epiphyseal ossification was possibly 6–8 yr at the time of demise (Fig. 1). This suggested age would represent either a profound new form of dwarfism or a fetus with premature ossification as the root causes for the “advanced bone age” phenotype (www.sciencemag.org/news/2013/05/bizarre-6-inch-skeleton-shown-be-human).

**Figure 1.**
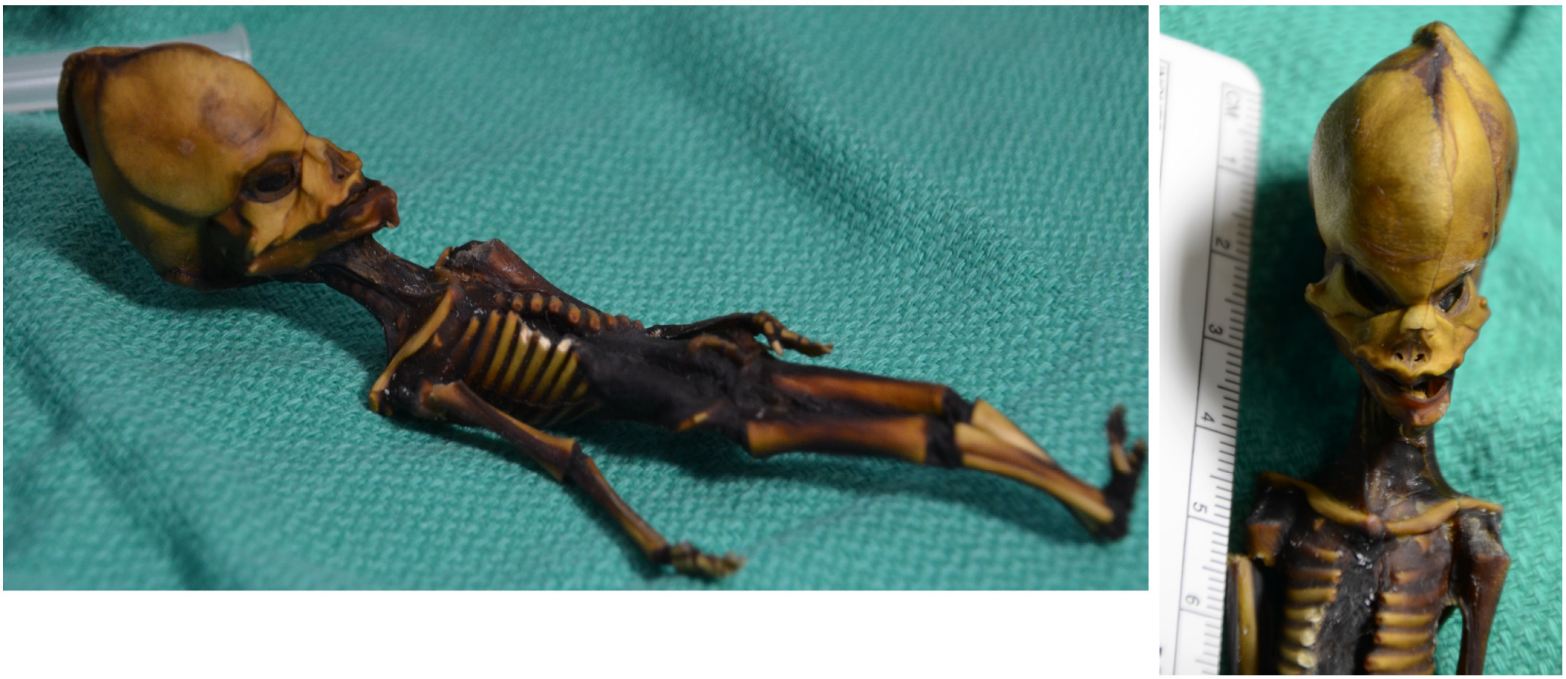
Mummified specimen from the Atacama region of Chile. Representative photograph of the 6-in skeleton (*left*) and frontal view of the skull of the Ata specimen (*right*). Picture courtesy E. Smith.

To date, the genetic drivers of the complex Ata phenotype have not been described with molecular evidence. Here, we present the first detailed whole-genome analysis of Ata, including genetic ancestry and sex determination, identification of disease- and phenotype-associated genes, and novel single nucleotide variation (SNV) detection.

## Results

We purified genomic DNA isolated from bone marrow and then performed whole-genome sequencing using the Illumina's HiSeq platform with an average coverage of 11.5× of 101-bp paired-end reads. We analyzed the DNA sequence reads using the large-scale genomic sequencing and analysis platform developed by Bina Technologies (a wholly owned subsidiary of Roche Sequencing Solutions). Specifically, first by running the BWA-MEM (Li 2013) aligner on the platform, ∼97% of 377,333,714 reads passing Illumina's internal quality filtering were successfully mapped. Of those reads, 89.77% were uniquely mapped to the human reference genome with decoy sequence (hs37d5) obtained from the 1000 Genomes Project (The 1000 Genomes Project Consortium 2015), 7.03% were multiply mapped, and 3.20% were unmapped. The reasons for the lack of match can include artifacts generated during library preparation, low-quality reads from the instrument, or insufficient data to allow alignment against the human reference standard. Because of low coverage limitations, we applied strict filtering criteria to ensure high-quality data for downstream processing. The human origin of the specimen was further confirmed by aligning the reads to other nonhuman primates including chimpanzee (panTro4, 88.01% of reads uniquely mapped) and rhesus macaque (rheMac3, 64.79% of reads uniquely mapped), indicating that the sample is more closely related to human than to other nonhuman primates (Supplemental Table S1).

In addition, we examined the mapping coverage of the Human Accelerated Regions (HARs) (Pollard et al. 2006; Hubisz and Pollard 2014) in the human genome that are conserved throughout vertebrate evolution but are strikingly different in humans. The five most accelerated HARs (HAR1–HAR5) were present in the Ata genome, with an average coverage (DP) of 12.6, 8.8, 8.9, 11.1, and 12.7, respectively (with reads mapping quality greater than 30). The distribution of the average coverage for the HARs set of 2701 regions had a mean value of 11.4 and standard deviation of 3.3. This indicates the HARs in the Ata genome had a coverage close to the average sequencing coverage, further confirming its human origin.

The mapped reads were further investigated to identify the type of variants observed in the Ata genome using the Genome Analysis Toolkit (GATK) suite on Bina's platform. In total, there were 3,356,569 SNVs detected, and 2,736,981 passed GATK Variant Quality Score Recalibration (VQSR), among which 96.44% were in the Database of Single Nucleotide Polymorphisms (dbSNP Build 147; http://www.ncbi.nlm.nih.gov/SNP/). The SNVs with passing qualities had a heterozygote to homozygote (het/hom) call ratio of 1.11, and the ratio of transitions to transversions (Ti/Tv) was 2.04. There were 518,365 indels detected, 401,822 of which passed VQSR and 91.48% of which were in dbSNP147. The passing indels showed a het/hom ratio of 1.06 and an insertion to deletion ratio of 0.89. In total, 6401 structural variations (SV) were detected with 1047 of passing quality, comprising 441 deletions, 525 duplications, 69 insertions, and 12 inversions. No whole chromosomal duplications or deletions were detected (Supplemental Table S2).

Preserved DNA extracts may exhibit DNA damage or contaminants. We characterized the extent and type of DNA damage present by measuring nucleotide misincorporations, particularly cytosine deamination at the ends of fragments. We observed a very small increase in the frequency of C → T and G → A substitutions resulting from cytosine deamination at the 5′ and 3′ ends, respectively, with an approximately twofold difference in substitution frequency at the ends of the read versus the center (mapDamage v2.0.2-14) (Supplemental Fig. S1; Jonsson et al. 2013; Methods). We applied a stringent quality filter during the mapping to trim the parts of the reads that look to contain damage. We also examined the contamination rate of the Ata DNA by assessing mitochondrial heterozygosity, as mitochondrial genomes are maternally inherited without recombination and contaminant-free DNA ought to exhibit little heterozygosity. Moreover, mtDNA is more stable over time and conditions, so we believe assessing the mtDNA for contamination is the best estimation in this case. The predicted probability of authenticity for Ata DNA was approximately 1.00, demonstrating little to no mitochondrial heterozygosity (contamMix v1.0-10) (Supplemental Fig. S2; Fu et al. 2014).

Taken together with the DNA damage results above, this indicates that the Ata DNA was relatively free of DNA damage and contaminants. Moreover, the average DNA fragment size for Ata is ∼300 bp which, based on a DNA-decay model (Allentoft et al. 2012), is consistent with a sample younger than 500 yr.

**Figure 2.**
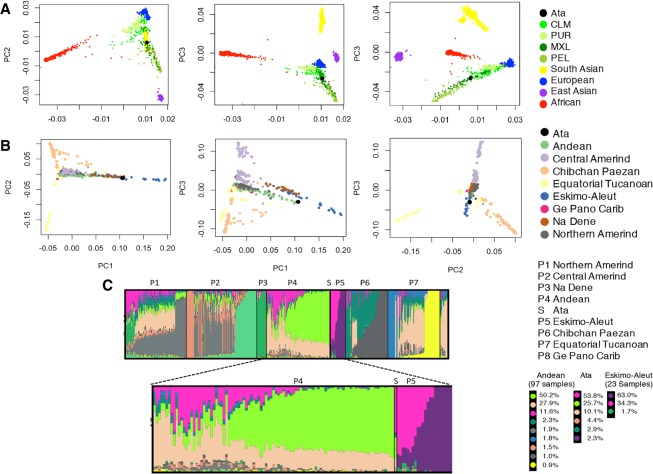
Genetic affinities of Ata specimen to the reference populations. (*A*) Scatterplot showing the three-dimensional PCA of five super populations of the 1000 Genomes Project (phase 3) and the Ata genome (black dot). Ata genome lies in the range of admixed populations and it is closest to Mexican ancestry from Los Angeles (MXL). (*B*) Three-dimensional PCA of eight major families from 52 Native American populations (493 individuals) and the Ata genome. The Ata genome is closest to the three individuals of Andean ancestry (in green), specifically belonging to the Chilote population, which supports a Chilean origin of the individual. (*C*) ADMIXTURE analysis with the Native American population data set. Global ancestry components of major Native American population group (P1–P8) and Ata (S) as identified by ADMIXTURE program at *K*****=****12. The magnified view of admixture mapping of Andean (P4), Ata (S), and Eskimo-Aleut (P5) samples suggests that Ata is an admixed individual with traces of European (in pink) and Native American ancestry with Andean lineage (in green and beige).

To assess the genetic ancestry of Ata specimen, the genotype data were merged into a reference set of five super populations of the 1000 Genomes Project (phase 3) using single nucleotide polymorphism sites (dbSNP v147) present in the Ata genome. We conducted a Principal Component Analysis (PCA) on the merged set of 3,974,633 SNPs and found that the Ata specimen lay in the range of admixed populations closest to Mexican ancestry from Los Angeles, USA (MXL); Colombians from Medellin, Colombia (CLM); and Peruvians from Lima, Peru (PEL) populations. These results suggest that the specimen was of likely of South American origin (Fig. 2A). Furthermore, an additional PCA was performed on the merged set of 363,969 SNPs from the Ata genome and a reference set of 52 Native American populations (Reich et al. 2012), which resulted in eight distinct population clusters (Methods). The PCA analysis demonstrated that Ata was in closest proximity to three individuals from the Andean region belonging to the Chilean Chilote population, further refining the ancestry of Ata to be of Chilean origin (Fig. 2B).

To further explore Ata's genetic ancestry, we estimated ancestry proportions, using the model-based population structure analysis implemented in the ADMIXTURE program (Alexander et al. 2009). This analysis suggests that Ata is admixed with a large proportion of European (an average 58%), East Asian (an average 25%), and other minor populations. This further confirms that Ata is a modern human specimen with a range of complex admixture events. Further, we estimated the genetic ancestry of Ata in reference to Native American populations and consistently observed an admixed genome with 53.8% European ancestry (pink) and a contribution of Native components (25.7% in green and 10% in beige) of Andean origin (Fig. 2C; Supplemental Fig. S4). These results are consistent with the ancestry estimations in Chilean individuals based on migration events in pre- and post-colonial periods reported by other groups (Reich et al. 2012; Homburger et al. 2015).

To examine the genetic determinants for Ata's unique phenotype, we investigated the sex of the individual, as these findings might be attributable to sex-linked disease. We used a sex determination technique that incorporated the ratio of sequence alignment to the Y (0.25×, including multiply mapped reads) and X Chromosomes (11.54×, requiring as few as 10^4^–10^5^ sequences) and the cytosine deamination signature of ancient DNA (Skoglund et al. 2013). The Ata specimen showed a very small fraction of alignment to the Y Chromosome with an *R*_Y_ of 0.0018, within the bound of 95% confidence interval for the inferred sex type “XX.” We also observed that there is no single read mapped to the *SRY* gene region on Chromosome Y. Together, these findings led us to infer that the Ata was female with two X Chromosomes.

To identify candidate genes with variants likely to be associated with disease, we prioritized functionally important gene variants from more than 2.7 million good-quality SNV with a stepwise reductionist approach using the ANNOVAR pipeline (Wang et al. 2010). In short, we filtered for nonsynonymous and splicing exonic variants, as well as segmental duplication regions; preserved variants in conserved genomic regions; and removed common variants (MAF >0.01) in the 1000 Genomes Project (Methods). Furthermore, variants believed to be likely benign by SIFT (Kumar et al. 2009; Sim et al. 2012) or PolyPhen-2 (Adzhubei et al. 2010) were removed. After applying a series of filtering procedures, we identified 64 coding region SNVs (nonsynonymous/stop-gain) predicted to be deleterious or possibly damaging with gene-based functional annotation (Supplemental Table S3).

Using the whole genome as a reference set for hypergeometric-based enrichment testing, we performed phenotype enrichment analysis for these 64 SNVs using the Human Phenotype Ontology database (HPO) (Köhler et al. 2014). In accord with Ata's peculiar anatomy, we found that the majority of these HPO-defined conditions were bone-associated, such as “Proportionate short stature” and “11 pairs of ribs” (Table 1; Supplemental Fig. S3). We also performed disease enrichment on these 64 exonic SNVs by interrogating the PharmGKB (Whirl-Carrillo et al. 2012) database using WebGestalt (Wang et al. 2013). The diseases identified were mostly associated to bone disorders, including scoliosis, Ehlers-Danlos syndrome, and musculoskeletal abnormalities (Table 2). We have also identified other potential deleterious variants in genes associated with dwarfism and osteochondrodysplasias in Ata genome (results not shown). As a negative control, we ran similar analyses on a randomly selected Peruvian female (HG01927) from the Native American population genome cohort in the 1000 Genomes Project. There were no overlapping genes with mutations identified in the Ata genome present in this individual. Furthermore, the enrichment analyses also did not yield any enrichment for genes associated with a disease or phenotype similar to Ata in this individual (Supplemental Table S7).

**Table 1.**
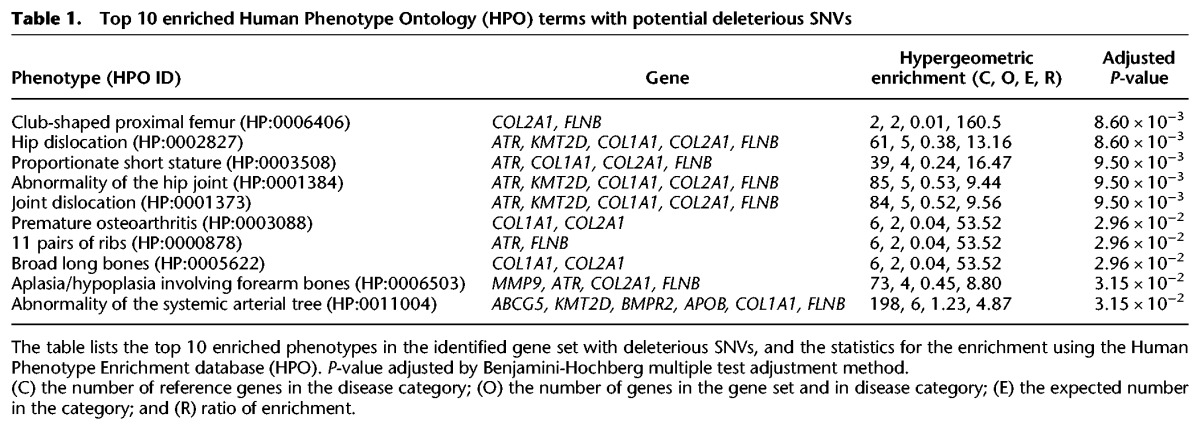
Top 10 enriched Human Phenotype Ontology (HPO) terms with potential deleterious SNVs

**Table 2.**
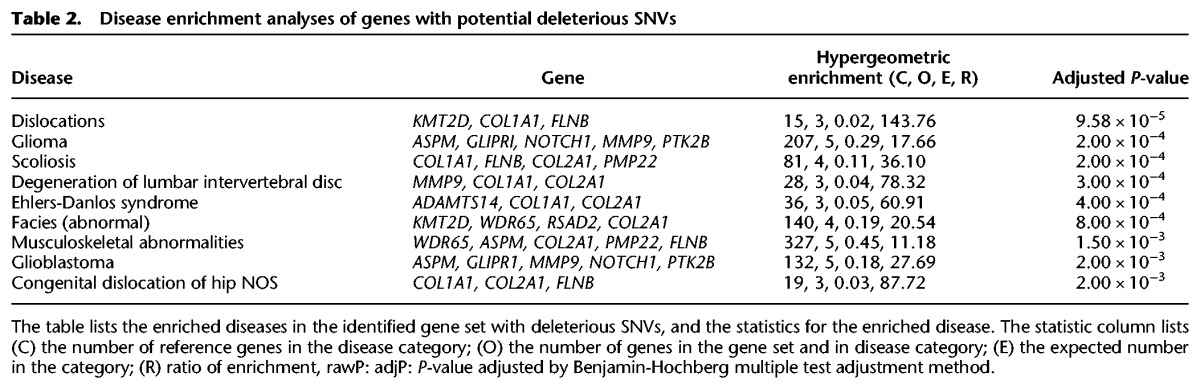
Disease enrichment analyses of genes with potential deleterious SNVs

At the gene sequence level, we identified four novel missense SNVs that had not been previously described. We found two rare SNVs (rs575285203, rs768451951) in genes encoding collagen (*COL1A1* and *COL2A1*); we found novel variants in filamin B (*FLNB*), lysine-specific methyltransferase (*KMT2D*, previously known as *MLL2*), thyroid hormone receptor interactor 11 (*TRIP11*), ataxia telangiectasia and Rad3-related protein (*ATR*), and a missense variant (rs2070426) in pericentrin (*PCNT*). These novel SNVs were predicted to be potentially damaging according to in silico functional prediction algorithms (MutationTaster [Schwarz et al. 2010], SIFT, or PolyPhen-2) available through dbNSFP (database for nonsynonymous SNPs’ functional predictions) (Liu et al. 2011) and SnpEff (Table 3; Supplemental Table S5; Cingolani et al. 2012). The MutationTaster score ranges from 0 to 1, and a larger score means higher accuracy for predicting functional alteration.

**Table 3.**
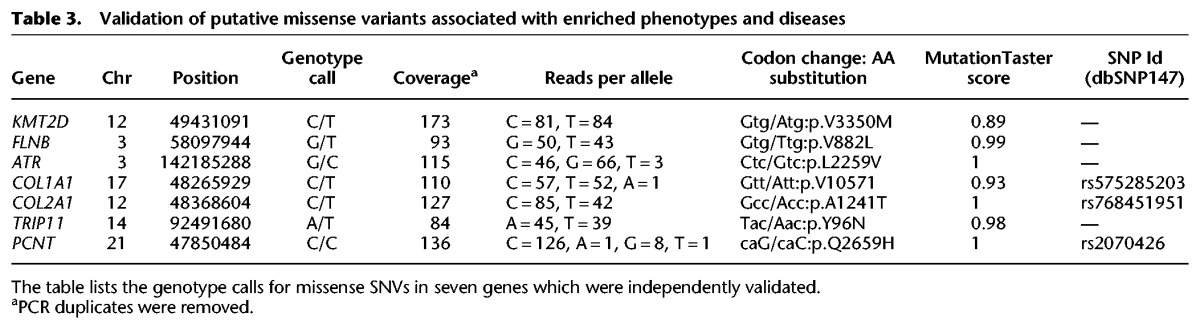
Validation of putative missense variants associated with enriched phenotypes and diseases

We confirmed the sequences of these SNVs with targeted capture of seven regions of the genome, each consisting of ∼800 bps centered on the candidate variants (Methods). We confirmed the heterozygous genotype calls for six of the seven SNVs (*COL1A1*, *COL2A1*, *FLNB*, *KMT2D*, *ATR*, and *TRIP11*). Additionally, we identified one locus in the *PCNT* gene that was originally called as heterozygous, but upon validation was determined to be homozygous for the nonreference, disease-associated allele (Table 3; Supplemental Table S5). These validation studies corroborated the SNVs identified in the Ata genome by the whole-genome sequencing method.

We also identified 557 indels (<50 bp size) in the coding region of Ata's genome following the same procedure applied for SNV detection. Further, breakdown of indels resulted in 257 frameshift variants (92 insertions and 165 deletions) and 223 non-frameshift variants (109 insertions and 104 deletions) in the genome. Additionally, we found nine genes with stop-gain and one gene with stop-loss mutations. We identified collagen catabolic process (GO:0030574) associated genes (*COL1A1*, *COL6A5*, *COL18A1*, *ADAMTS2*) with frameshift deletions. In addition, there were frameshift deletions found in Histocompatibility complex C1-set domain containing genes *HLA-B*, *HLA-DQA1*, *HLA-DRB1*.

We next examined the structural variation in Ata genome. There were in total 132 exonic structural variation (SV) with 128, 4, and 2 duplications, deletions, and inversions, respectively. We identified duplication in the genes *USP17* and *USP18* associated with protein deubiquitination (GO:0016579) spans 4–25 kb on Chromosome 4p16.1. There were four deletions in olfactory receptor genes (*OR52N1*, 24.7 kb and *OR52N5,* 0.31 kb) on Chromosome 11, *OVCH2* (0.31 kb) on Chromosome 15, and *LILRA3* (6.8 kb) on Chromosome 19. The genes *SYT6* and *CNTN5* include 0.825- and 8.254-kb inversions, respectively. No translocations were detected in the genome. There was not sufficient evidence with precise annotation based on known expression or copy number variation to link the structural variants to the observed phenotype (Supplemental Table S8).

## Discussion

Our findings demonstrate that whole-genome sequencing can be readily applied to the analysis of archeologically and anthropologically relevant individual human specimens with genetic disorders of unknown origin. Current databases are now sufficiently detailed to provide clues as to ancestry as well as health outcomes for patients—even specimens with dramatic claims that would dissuade such samples from serious inquiry. Notably, we identified several novel mutations in genes that would be predictive of the skeletal malformations exhibited in Ata.

In the Ata specimen, we have identified known mutations in genes associated with disease such as cranioectodermal dysplasia (Beck et al. 2015) and Greenberg skeletal dysplasia, which each produce phenotypes similar to that observed in the Ata specimen. Ata's genome also contained previously reported variants (rs41298151, p.Gly465Ala) in *FREM1* and *FLNB* (rs1131356, p.Asp1157Asn), which are associated with congenital diaphragmatic hernia (Walczak-Sztulpa et al. 2010), a relatively common, life-threatening birth defect in which the diaphragm does not develop properly (Supplemental Table S6; Stenson et al. 2014).

The SNVs that we identified are novel, but previously identified and distinct mutations in all seven genes are implicated in osteochondrodysplasias and represent plausible causes of Ata's abnormal skull morphology, small stature, 10 ribs, and premature bone age. Type 1 collagen (*COL1A1*) and Type II collagen (*COL2A1*) are major structural proteins of bone and cartilage, respectively. Autosomal dominant mutations in *COL1A1* are known causes of Ehlers-Danlos syndrome and osteoporosis (Steiner et al. 1993; Byers 2000). Similarly, autosomal dominant mutations in *COL2A1* are responsible for a number of osteochondrodysplasias (Barat-Houari et al. 2016), and mutations in both *COL2A1* and *TRIP11* are implicated in Type 2 and Type 1A achondrogenesis, respectively. *KMT2D* mutations are known to be associated with Kabuki syndrome, which is characterized by cranial and facial malformations, growth deficiency, short stature, and skeletal malformations. Mutations in *ATR* can cause Seckel syndrome 1, which is characterized by mental retardation and proportional dwarfism (Griffith et al. 2008). More than 30 described mutations in *PCNT* cause failure in centrosome division, resulting in microcephalic osteodysplastic primordial dwarfism type II (MOPDII), which is characterized by short bones and microcephaly. *FLNB* is important for fetal skeletal development and *FLNB* mutations are associated with atelosteogenesis I, Larsen syndrome, and spondylocarpotarsal synostosis syndrome, a disease of ectopic ossification that causes inappropriate fusion of the bones of the vertebrae, wrists, and ankles. Further, our findings are consistent with the previously reported skeletal dysplasia disease genes in the nosology and classification of genetic skeletal disorders (Bonafe et al. 2015).

Moreover, we found a report on a consanguineous family with two affected children diagnosed with a most severe form of osteogenesis imperfecta (i.e., short stature, low bone density, and severe vertebral compression fractures) in the first years of life harboring 19 rare homozygous and compound heterozygous mutations. These findings further confirm that a number of rare variants enriched in known skeletal phenotypes associated with Ata is consistent with other studies, and suggests that some carried sublethal mutations from one or more of the parents might have resulted in the unusual observed phenotype (Fahiminiya et al. 2013).

The higher susceptibility to induce human phenotypic variation and disease due to deletions and structural variations in the human genome are expected. We identified a 4-bp frameshift deletion in *COL1A1* (Chr 17: 482,63858–482,63861, TCCAG>T) and a 6.8-kbp-large frameshift deletion in *LILRA3* (Chr 19: 548,00800–548,0760), which is consistent with the finding by other groups in patients with skeletal malformations and dysplasia (Chopra et al. 2015; Bae et al. 2016). The combination of single base substitutions and frameshift deletions detected in collagen genes might be playing a major role in the abnormality of body structure and developmental disorders. Further, deep sequencing of the genome might reveal other phenotype-associated structural variations that are limited in the current analyses due to low coverage of the genome.

Taken together, it is entirely plausible that the chance combination of multiple known mutations and novel SNVs identified here may explain Ata's small stature, inappropriate rib count, abnormal cranial features, and perceived advanced bone age. Given the size of the specimen and the severity of the mutations described above, it seems likely the specimen was a preterm birth. Although we can only speculate as to the cause for multiple mutations in Ata's genome, the specimen was found in La Noria, one of the Atacama Desert's many abandoned nitrate mining towns, which suggests a possible role for prenatal nitrate exposure leading to DNA damage (Andreassi et al. 2001).

Although the extraordinary phenotype of the specimen drove broad discussion as to its origin (*Sirius*), and no hypothesis was left off the table during analysis, the specimen is shown here to have a purely earthly origin with mutations that reflect the visual determinations. Indeed, although purely speculative, the premature ossification phenotype observed here might eventually be understood to be a process that could be (medically) manipulated in bone development. Future studies should investigate the novel sequence variations that we present here, which will require molecular characterization of individual mutations and comparisons with other ethnically focused whole-genome sequencing databases and that can contribute to determining causal-effect relationships both at the molecular and population levels.

## Methods

### Sample collection

The specimen was scanned with X-ray analysis to identify bone dysmorphias. Scanning revealed where bone marrow sample could be readily isolated (with minimal damage to the specimen) from the ribs and right humerus of the specimen with a set of surgical instruments in a designated sterile area zone following sterile aseptic technique. Some inner skull scrapings, bone material, and dura mater were also recovered during the procedure.

### DNA isolation

DNA was extracted using High Pure Viral Nucleic Acid Large Volume Kit (Roche Diagnostics; Cat. 05 114 403 001), with some modifications. Briefly, bone's fragments were ground, and the powder was resuspended in 1 mL of binding buffer supplemented with poly(A) carrier RNA and proteinase K. Samples were incubated overnight at 37°C. The subsequent steps were performed according to the manufacturer's instructions.

### DNA library preparation

The Illumina's TruSeq indexed pair-ended DNA library preparation protocol was performed automatically on the SPRIworks system (Beckman Coulter). By using cartridge and method card specific to Illumina sequencing system, a fragment library can be prepared for Illumina sequencers. After individual libraries were constructed, qualities and band-sizes were assessed using Bioanalyzer High Sensitivity Chip (Agilent Technologies) and Qubit (Life Technologies). Libraries were also quantified by qPCR using the Library Quantification Kit for Illumina sequencing platforms (KAPA Biosystems), using an ABI 7900HT Real-Time PCR System (Life Technologies). Libraries were normalized to a working concentration of 10 nM, using the molarity calculated from qPCR and adjusted for fragment size with the Bioanalyzer analysis. They were finally sequenced on Illumina's MiSeq and HiSeq 2000 (Supplemental Table S4). All raw FASTQ files were extracted for sequencing alignment and further analysis.

### Whole-genome analysis

More than 377 million 101-bp paired-end reads (on average 11.5× coverage) sequenced from the genomic DNA of the specimen were processed with the Bina's read alignment, variant calling, and expression module (RAVE) (1.5.0-dev-217-ga8038cc). Bina's RAVE performed secondary analysis on the NGS data, which included sequence alignment, small variant calling, and structural variation (SV) as well as copy number variation (CNV) detection, following the best practices for secondary sequence analysis recommended by the Broad Institute, where appropriate.

Specifically, the Bina's in-memory sorter was used concurrently with alignment to minimize latency; BWA-MEM (Li 2013) v0.7.5a was used for sequence alignment; GATK (DePristo et al. 2011) v2.8 with HaplotyperCaller and VQSR was used for SNV and small indel detection and filtering; and MetaSV (Mohiyuddin et al. 2015) was used to integrate different SV/CNV signals detected by four orthogonal algorithms, i.e., detection of signals using read depths by CNVnator (Abyzov et al. 2011), split-reads by Pindel (Ye et al. 2009), paired-end reads by BreakDancer (Chen et al. 2009), and junctions by BreakSeq (Lam et al. 2010). The integrated call set was annotated with confidence labels (PASS/LowQual) and detection methods by MetaSV. Small variants were then passed to the Bina's annotation and analytics intelligence module (AAiM) (v0.1.6), which uses technologies such as Hadoop and HBase for fast, multitype variants annotation. Bina's AAiM also provides various real-time filtration and intersection with more than 100 annotation features from databases such as RefSeq and HGMD.

### Genetic ancestry determination

Ata's genome sequence was mapped onto the Native American population data set (364,470 SNPs genotyped in 493 samples from 52 Native American populations) (Reich et al. 2012). GATK UnifiedGenotyper (McKenna et al. 2010; DePristo et al. 2011) was used to retrieve the genotypes of matched 364,470 SNP positions from Ata genome. SNPs with fewer than five sequence reads were marked as incomplete genotypes. After removing triallelic and inconsistent SNPs by merging the two data sets, 363,969 SNPs were retained for the downstream PCA analysis. Principal component analysis (PCA) was performed using smartpca (Price et al. 2006) implemented in the EIGENSOFT package v5.0.1.

Further, the Ata genome was mapped on the 1000 Genomes Project phase (1KGP) 3 integrated_v2 data set, with 77,233,099 autosomal SNPs identified. Similarly, GATK UnifiedGenotyper retrieved all the SNP genotypes from the Ata genome. Those SNPs with minor allele frequency <0.05 and Hardy-Weinberg equilibrium *P*-value <0.00001 were removed with PLINK (Purcell et al. 2007), and linkage disequilibrium (LD) pruning was performed based on the variance inflation factor (plink-indep 50 5 2). After removing triallelic and inconsistent SNPs by merging the two data sets, 3,974,633 SNPs were retained for the downstream PCA analysis.

We next performed ADMIXTURE (Alexander et al. 2009) analysis on the merged data sets as described above for PCA analyses on the unmasked Native American samples and the 1000 Genome Project panel. ADMIXTURE models were explored at varying number of *K* clusters with cross-validation for *K* = 6 through *K* = 12 with 10 replicates for each *K* with random seed for local ancestry estimation. We used a default block relaxation algorithm for the method optimization. The same analyses were repeated at *K* = 4 and replicated 10 times with random seed for global ancestry estimation (Supplemental Fig S5). The postprocessing clustering inference (Q matrices) from ADMIXTURE program were parsed into Pong cluster visualization tool for analyzing and visualizing membership in latent clusters with a native interactive D3.js visualization (Behr et al. 2016).

### Contamination and DNA damage estimates

To determine the degree to which the Ata specimen was affected by DNA damage, we used the mapDamage v2.0.2-14 program (Jonsson et al. 2013) to measure the rate of C → T and G → A substitutions at the 5′ and 3′ ends of the read fragments. We found an approximate twofold increase in the rate of deamination for the ends of the reads compared to the center (C → T substitution rate = 0.0184 versus 0.0081 and G → A substitution rate = 0.0267 vs 0.0112). Overall, we did not observe much damage in Ata's genome; as a result, the UNG treatment prior to amplification was not recommended. We used a stringent quality filter during the mapping to trim the parts of the reads that look to contain damage. We then used contamMix v1.0-10 to estimate contamination using rate of mitochondrial heterozygosity. This analysis predicted the probability of authenticity of the specimen to be ∼1.00, due to limited to no heterozygosity of the mitochondria.

### Sex determination by chromosomal sequence alignment

To determine the sex of the Ata specimen, the read depth on the sex chromosomes were analyzed. The Y PARs were masked out by “N” in the reference genome (hs37d5); therefore, the X PARs can be treated as diploid even for male samples. The Chromosome Y is showing a significantly lower coverage than Chromosome X (0.25× versus 11.54×). Approximately 70% of these reads mapped to Chromosome Y are with mapping quality zero, which means they can be mapped to multiple locations either on Chromosome Y or other chromosomes. Only 17% of these reads have mapping quality higher than or equal to 30, and ∼50% of these reads with mapping quality ≥30 had soft clipping bases.

Following previous guidelines (Skoglund et al. 2013), we calculated the fraction sequences aligned to the Y Chromosome, which is a ratio of the total number of sequences aligned to either sex chromosome (*R*_Y_). The Ata specimen showed an *R*_Y_ of 0.0018, within the bound of 95% confidence interval, and is assigned an inferred sex of “XX.” Because the specimen is female, we were not able to use Y Chromosomal heterozygosity to make estimates of contamination with nuclear DNA.

For benchmarking, we also examined the reads aligned to the sex chromosomes for the well-characterized female genome NA12878 recently sequenced again by the National Institute of Standards (NIST). This sample had roughly 49.1× coverage across the whole genome and 0.497× coverage on the Y Chromosome. It showed an *R*_Y_ of 0.0016 with inferred sex type “XX.”

### Prioritizing the candidate variants by ANNOVAR pipeline

We used a customized filtering procedure from ANNOVAR (Wang et al. 2010) pipeline to identify a subset of SNVs after passing the QC threshold from Bina's variant detection method (“Whole-genome analysis” section). This is a stepwise variant- and gene-level annotation-based filtering scheme to identify candidate genes with potential variants likely to be associated with disease. The filtering steps include (1) identifying nonsynonymous and splicing variants; (2) removing variants in segmental duplication regions; (3) keeping variants in conserved genomic regions based on 46-way alignment; (4) removing common variants observed in the 1KGP (the October 2014 release) for European, Asian, and African populations, and National Heart, Lung, and Blood Institute (NHLBI) Exome Sequencing Project (ESP 6500, http:// esp.gs.washington.edu/) for European and African populations; and (5) removing variants observed in The NCBI Short Genetic Variations database (dbSNP, http://www.ncbi.nlm.nih.gov/SNP, version 138).

### Enrichment analysis

The list of candidate SNVs identified by ANNOVAR pipeline were analyzed using the WebGestalt (Wang et al. 2013) enrichment analysis tool. The list of SNVs, indels, and SVs were annotated against the PharmGKB (Whirl-Carrillo et al. 2012) and Human Phenotype Ontology (HPO) (Köhler et al. 2014) databases for disease and phenotype enrichment, respectively, with the whole genome as a reference set for hypergeometric test for enrichment. The *P*-values for enrichment were adjusted by the Benjamini-Hochberg method for multiple hypotheses correction. We also ran gene ontology (GO; www.geneontology.org) enrichment analyses on indels and SVs.

### Sequence validation

Because the average fragment size of the extracted DNA was ∼300 bp, we were unable to predict binding sites for Sanger sequencing primers that would reliably amplify the regions surrounding the loci identified in our enrichment analysis in our fragmented sample. Instead, to validate the genotype calls for the variants found through our enrichment analysis, we generated targeted capture probes and performed resequencing of the loci. We first designed primers targeting an ∼800 bp fragment centered around the SNVs of interest and amplified the regions surrounding these loci using modern DNA from a Peruvian individual. The amplicons were fragmented to an average size of ∼100 bp using a Covaris S220 focused ultrasonicator, and in-vitro transcription was performed using the AmpliScribe T7-Flash Biotin-RNA Transcription kit to produce biotinylated RNA probes targeting the regions of interest. Capture was performed as previously described (Carpenter et al. 2013). The captured fragments were then sequenced in a 76-bp paired-end mid-output run on a NextSeq 500 instrument, to an average coverage of 120× per SNV.

## Data access

The whole-genome sequence data from this study have been submitted to the Sequence Read Archive (SRA; https://www.ncbi.nlm.nih.gov/sra/) under accession number SRP083100.

## Supplementary Material

Supplemental Material
