# Supplementary material for: Whole-genome sequencing of Atacama skeleton shows novel mutations linked with dysplasia
Source: Genome Res. 2018 Apr;28(4):423–31. doi: 10.1101/gr.223693.117 (PMC5880234; doi:10.1101/gr.223693.117)
Supplement: Supplemental Material [file supp_28_4_423__index.html]

Whole-genome sequencing of Atacama skeleton shows novel mutations linked with dysplasia — Supplemental Material 

# Whole-genome sequencing of Atacama skeleton shows novel mutations linked with dysplasia

## Supplemental Material

- Supplemental\_Table\_S8.xls
- Supplemental\_Table\_S7.xls
- Supplemental\_Table\_S6.xls
- Supplemental\_Table\_S5.xls
- Supplemental\_Table\_S4.xls
- Supplemental\_Table\_S3.xls
- Supplemental\_Table\_S2.xls
- Supplemental\_Table\_S1.xls
- Supplemental\_Fig\_S5.pdf
- Supplemental\_Fig\_S4.pdf
- Supplemental\_Fig\_S3.pdf
- Supplemental\_Fig\_S1.pdf
- Supplemental\_Fig\_S2.pdf
- Supplemental\_Note.docx
