# Supplementary material for: Whole-genome sequencing of Atacama skeleton shows novel mutations linked with dysplasia
Source: Genome Res. 2018 Apr;28(4):423–31. doi: 10.1101/gr.223693.117 (PMC5880234; doi:10.1101/gr.223693.117)
Supplement: Supplemental Material [file supp_gr.223693.117_Supplemental_Fig_S5.pdf]

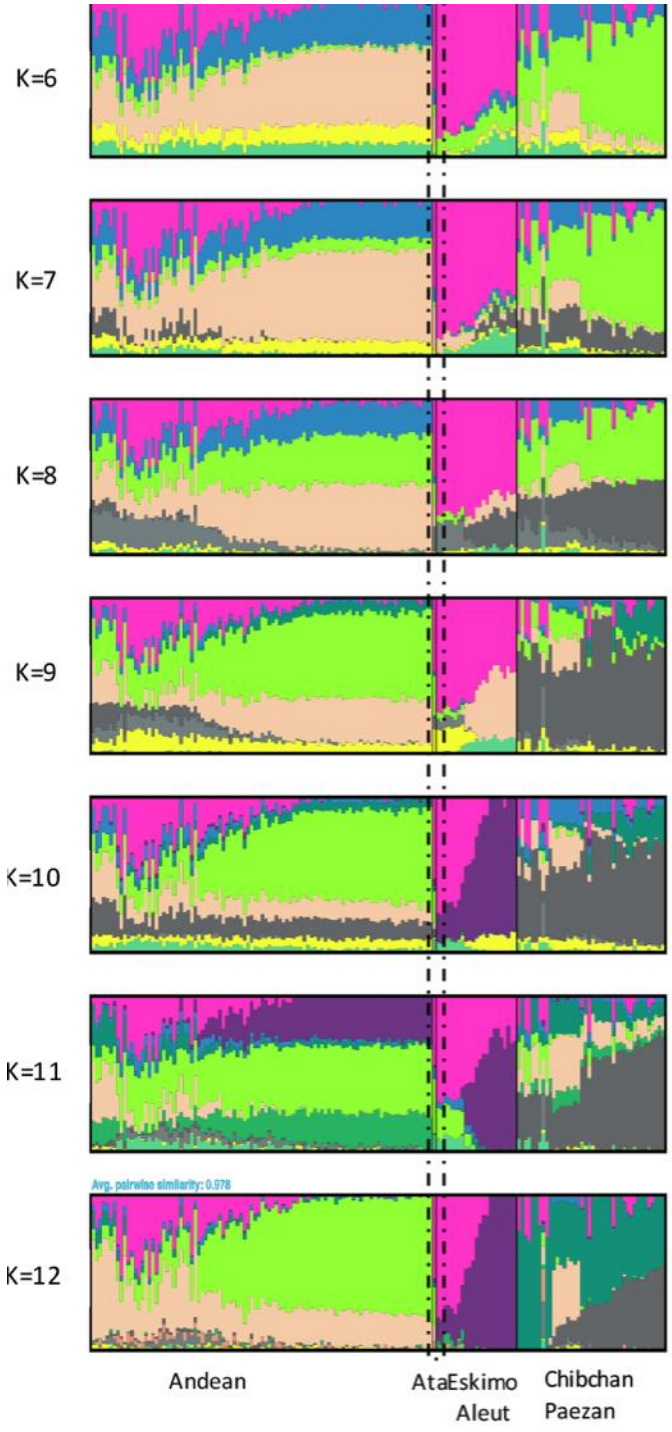

Supplemental Fig S5. ADMIXTURE analyses (K=6 through 10) for local ancestry estimation of Ata mapped on the unmasked 52 Native American populations dataset. This plot depicts the fractional memberships of Ata[box] in reference to Native American populations (Andean and Eskimo-Aleut and Chibchan Paezan population) with average pairwise similarity =0.978 across ten replicate runs at K=12.
