# Supplementary material for: Whole-genome sequencing of Atacama skeleton shows novel mutations linked with dysplasia
Source: Genome Res. 2018 Apr;28(4):423–31. doi: 10.1101/gr.223693.117 (PMC5880234; doi:10.1101/gr.223693.117)
Supplement: Supplemental Material [file supp_gr.223693.117_Supplemental_Fig_S4.pdf]

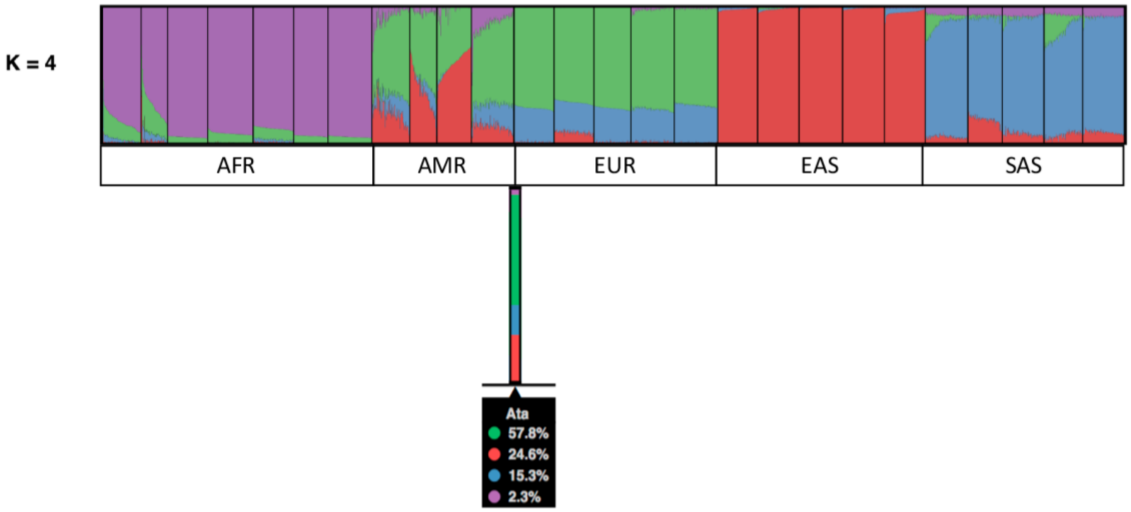

Supplemental Fig S4. ADMIXTURE analyses (K=4) for global ancestry estimation in a model-based manner from autosomal SNPs in Ata matched with 1000 Genomes phase 3 panel reference population. This plot depicts the mode of major human continental groups (AFR, AMR, EUR, EAS AND SAS) separated by dark black solid line. We show that Ata is an admixed individual with large fraction of European ancestry (in green), East and South Asian ancestry (in red and blue respectively) and a tiny fraction of African lineage (in violet).
