# Supplementary material for: Whole-genome sequencing of Atacama skeleton shows novel mutations linked with dysplasia
Source: Genome Res. 2018 Apr;28(4):423–31. doi: 10.1101/gr.223693.117 (PMC5880234; doi:10.1101/gr.223693.117)
Supplement: Supplemental Material [file supp_gr.223693.117_Supplemental_Fig_S1.pdf]

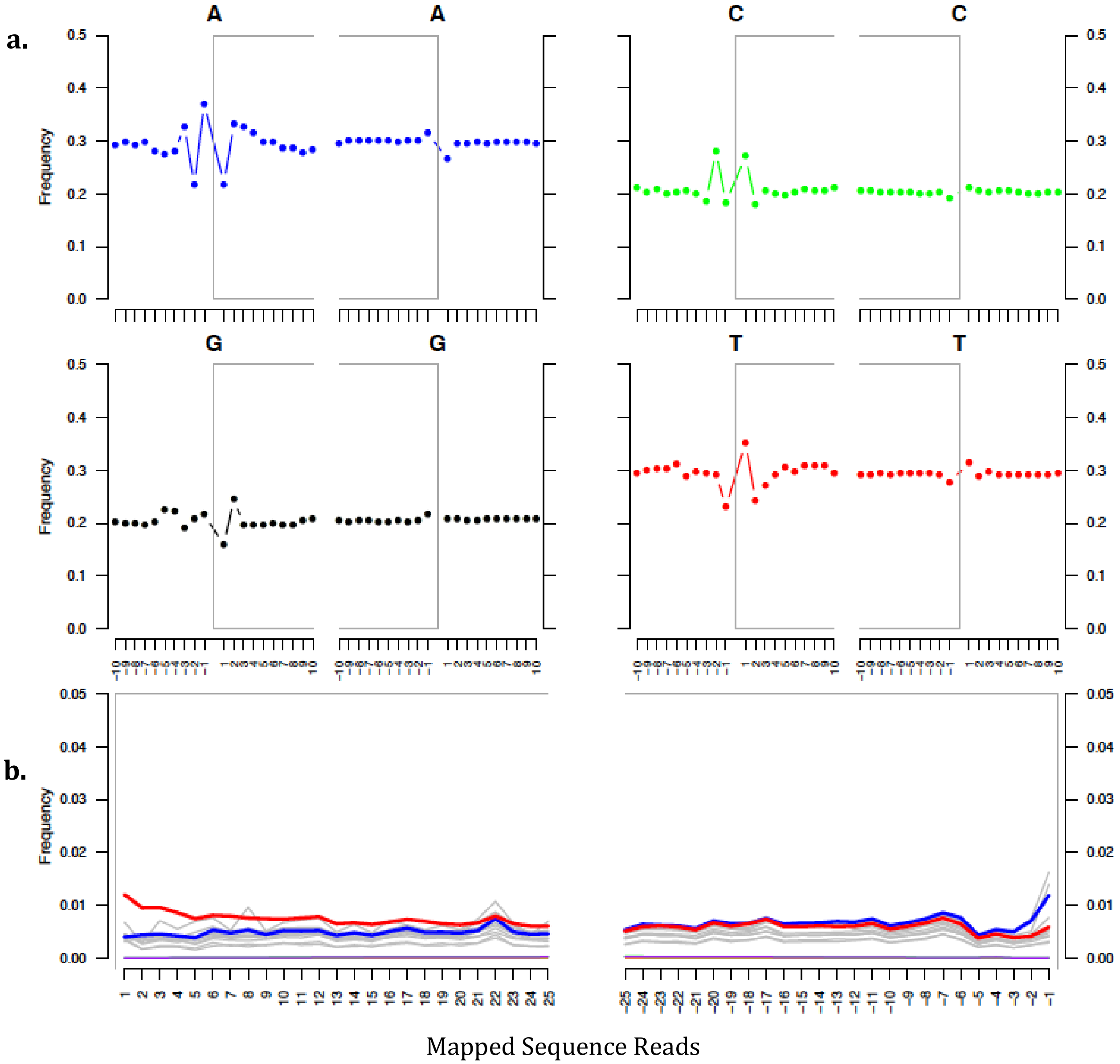

Supplemental Fig S1. mapDamage results for the Ata specimen. A. This plot illustrates frequency of each nucleotide from -10 to -1 (in the reference genome) to +1 to +10 (in sequenced reads) with grey brackets denoting the 5' and 3' paired ends of the sequenced reads. B. This plot indicates the positions' specific substitution rates from the 5' (left) and 3' (right) ends. Red is C->T substitutions, blue is G->A substitutions, and all possible misincorporations are plotted in grey.
