# Supplementary material for: Whole-genome sequencing of Atacama skeleton shows novel mutations linked with dysplasia
Source: Genome Res. 2018 Apr;28(4):423–31. doi: 10.1101/gr.223693.117 (PMC5880234; doi:10.1101/gr.223693.117)
Supplement: Supplemental Material [file supp_gr.223693.117_Supplemental_Fig_S2.pdf]

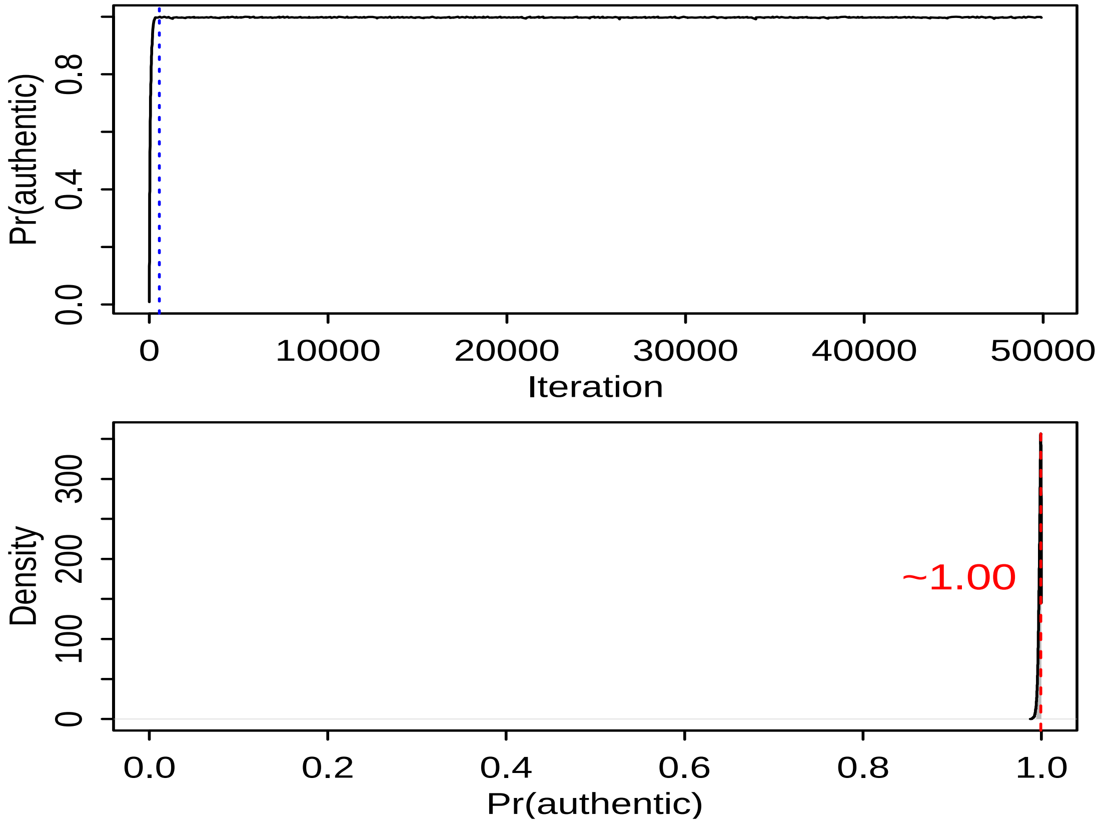

Supplemental Fig S2. ContamMix results from mitochondrial DNA of the Ata specimen. The top panel depicts the probability that the sample is authentic for each iteration of the algorithm. The bottom panel illustrates the density of probability values in the distribution.
