# Supplementary material for: Whole-genome sequencing of Atacama skeleton shows novel mutations linked with dysplasia
Source: Genome Res. 2018 Apr;28(4):423–31. doi: 10.1101/gr.223693.117 (PMC5880234; doi:10.1101/gr.223693.117)
Supplement: Supplemental Material [file supp_gr.223693.117_Supplemental_Note.docx]

Preliminary Report on the Atacama Specimen

In the Fall of 2012 a biomedical analysis was initiated of a mummified specimen claimed to have originated in the Atacama Desert of Chile, South America. High resolution photographic, X-Ray and Computed Tomography evidence was taken, along with purification of DNA for whole genome sequence (WGS).

The first stage of the study involved analysis by medical experts specializing in pediatric growth abnormalities, with primary expertise in the genetics of bone disorders. The objective of these initial studies was to rule out, or in, previously known syndromes or rare disorders that could explain the symptoms observed in the specimen. A second consideration was to determine the “age at time of death”—given that its size would suggest that the specimen was a pre-term fetus, stillborn, or a deformed post-natal child. A third, but important, consideration was to determine if the specimen was a non-human hominid such as a South American primate.

Morphologic features include that the specimen has only 10 ribs, mild mid face hypoplasia, and shows abnormalities of the skull. The observed abnormalities do not fall into any standard or rare classification of known human pediatric disorders. As represented by a specialist in pediatric human bone and growth disorders, the 6 inch specimen is a human that was likely 6-8 years of age at the time of death (age based on epiphyseal plate X- Ray density standards). X-Ray imaging and CT scan results confirmed the specimen is biological and is not a non-human primate. The specimen was concluded by the medical specialist to be a human child with an apparently severe form of dwarfism and other anomalies.

To further investigate the specimen, and to determine possible genetic drivers of its observed morphology, tissue from the specimen was subjected to whole genome sequencing. 3 milligrams of tissue was used to prepare 12.5 micrograms of purified genomic DNA. The DNA was of high quality, showing little to no serious degradation. DNA was subjected to Illumina library preparation and sequencing on Illumina Miseq (PE250x2), Genome Analyzer IIX (SR36x1), and Hiseq 2000 PE100x2) sequencing platforms according to manufacturer’s protocols.

Over 560 million paired end sequence reads passed automated quality control filters and provided an estimate 19.6X coverage for the whole genome. Approximately 509 million (~91%) reads were mapped to the human reference genome hg19 (providing a 17.7 fold coverage of the genome)

Reconstruction of the mitochondrial DNA sequence and analysis shows an allele frequency consistent with a B2 haplotype group found on the west coast of South America, supporting the claimed origination of the specimen from the Atacama Desert region of Chile. Sequence analysis definitively rules out the specimen as an example of a New World primate.

Preliminary results demonstrate no statistically relevant alterations of genes encoding proteins commonly associated with known genes for primordial dwarfism or other forms of dwarfism. Therefore, if there is a genetic basis for the symptoms observed in the specimen the casual mutation(s) are not apparent at this level of resolution and at this stage of the analysis.
