# Supplementary material for: Whole-genome sequencing of Atacama skeleton shows novel mutations linked with dysplasia
Source: Genome Res. 2018 Apr;28(4):423–31. doi: 10.1101/gr.223693.117 (PMC5880234; doi:10.1101/gr.223693.117)
Supplement: Supplemental Material [file supp_gr.223693.117_Supplemental_Fig_S3.pdf]

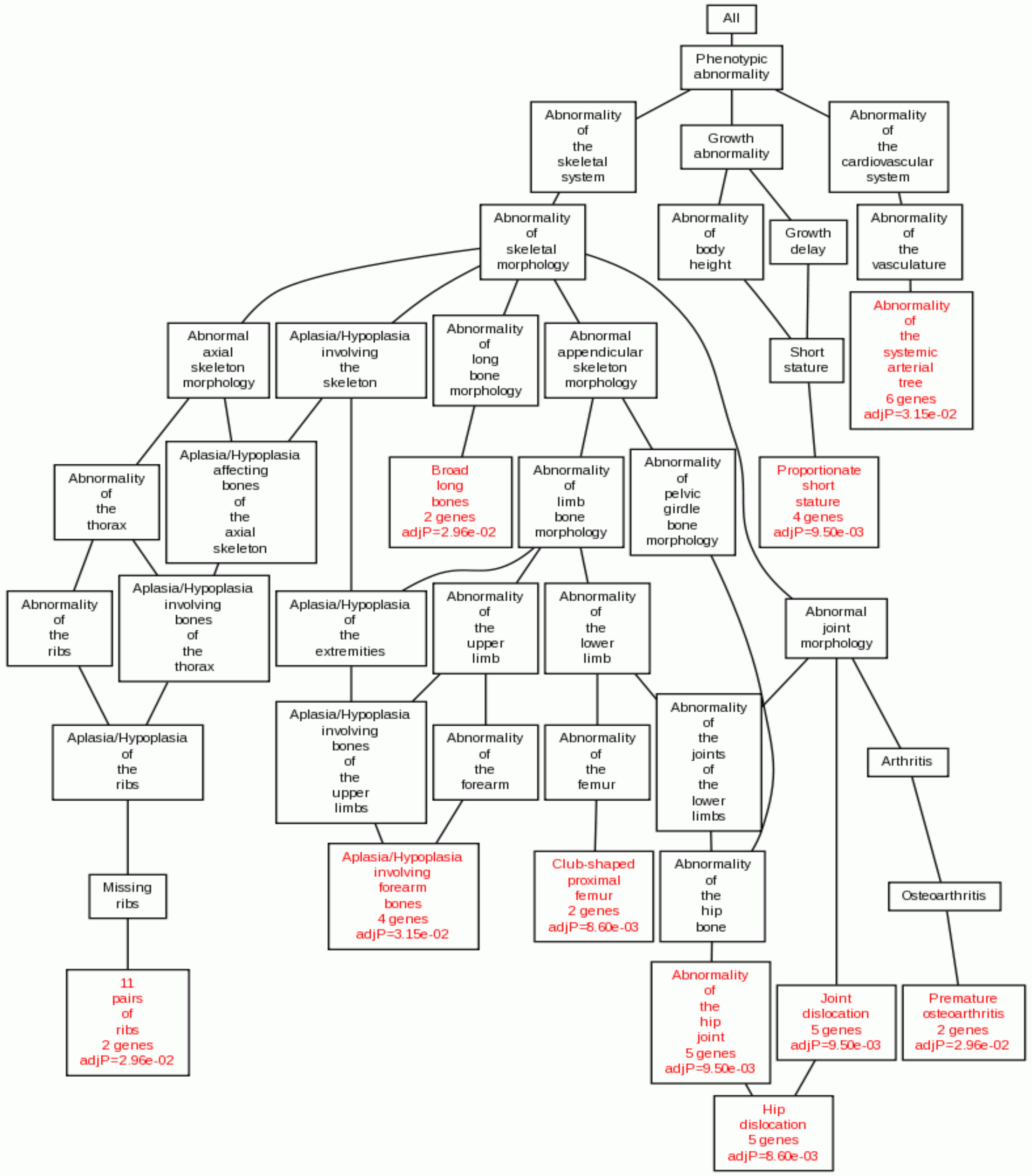

Supplemental Fig S3. Enriched phenotypes in the Ata specimen. The DAG illustrates the phenotype hierarchy terms from HPO. The boxes in light and dark red are the phenotypes significantly enriched with BH adjusted p-value ≤ 0.01 from the list of deleterious SNVs identified.
